# Resource availability modulates the effect of body size on reproductive development

**DOI:** 10.1002/ece3.9722

**Published:** 2023-01-06

**Authors:** Réka Gergely, Jácint Tökölyi

**Affiliations:** ^1^ MTA‐DE “Momentum” Ecology, Evolution and Developmental Biology Research Group, Department of Evolutionary Zoology University of Debrecen Debrecen Hungary; ^2^ Pál Juhász‐Nagy Doctoral School University of Debrecen Debrecen Hungary

**Keywords:** body size, food availability, reproduction, size manipulation, survival

## Abstract

Within‐species variation in animal body size predicts major differences in life history, for example, in reproductive development, fecundity, and even longevity. Purely from an energetic perspective, large size could entail larger energy reserves, fuelling different life functions, such as reproduction and survival (the “energy reserve” hypothesis). Conversely, larger body size could demand more energy for maintenance, and larger individuals might do worse in reproduction and survival under resource shortage (the “energy demand” hypothesis). Disentangling these alternative hypotheses is difficult because large size often correlates with better resource availability during growth, which could mask direct effects of body size on fitness traits. Here, we used experimental body size manipulation in the freshwater cnidarian *Hydra oligactis*, coupled with manipulation of resource (food) availability to separate direct effects of body size from resource availability on fitness traits (sexual development time, fecundity, and survival). We found significant interaction between body size and food availability in sexual development time in both males and females, such that large individuals responded less strongly to variation in resource availability. These results are consistent with an energy reserve effect of large size in *Hydra*. Surprisingly, the response was different in males and females: small and starved females delayed their reproduction, while small and starved males developed reproductive organs faster. In case of fecundity and survival, both size and food availability had significant effects, but we detected no interaction between them. Our observations suggest that in *Hydra*, small individuals are sensitive to fluctuations in resource availability, but these small individuals are able to adjust their reproductive development to maintain fitness.

## Graphical abstract

We attempted to untangle the body size—fitness trait correlations with *Hydra oligactis* model system, by grafting procedure to directly manipulate body size, and with food availability manipulation. With these treatments, we attempted to reveal the effects of body size, resource availability, and their interaction on reproduction and survival.

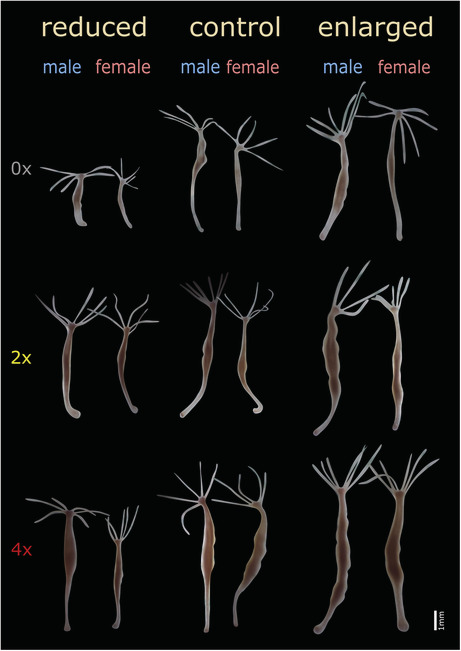

## 1 INTRODUCTION

A large proportion of life history traits' variation can be attributed to body size in mammals, reptiles, birds, and invertebrates (Lindstedt & Boyce, 1985). Body size is one of the most important quantitative traits in evolutionary ecology research because of its strong correlation with physiological and fitness characters (Blanckenhorn, 2000; Reiss, 1989; Roff, 1993). Body size varies considerably both within and among species and is affected by contrasting selective forces due to its complex effects on fitness components. For instance, large size can be under positive selection due to its association with higher fecundity, but selection could also favor early maturation, thus small size (Amarillo‐Suárez et al., 2011; Blanckenhorn, 2000; Harvey et al., 2006).

Purely from an energetic perspective, the correlation between body size and fitness traits could stem from several distinct mechanisms. First, large body size entails larger energy reserves, which can be allocated between different tasks (e.g., self‐maintenance, reproduction, survival; the “energy reserve” hypothesis) (Lindsey, 1966; Millar & Hickling, 1990; Roff, 1993; Stearns, 1992). This hypothesis predicts that larger animals will start to reproduce earlier with greater effort, resulting in higher reproduction, even in limited environments, because of the higher amount of accumulated energy (Lindstedt & Boyce, 1985; Reim et al., 2006). Body size positively affects fecundity in taxa as diverse as sponges (e.g., *Rhopaloeides odorabile*) (Whalan et al., 2007), cnidarians (e.g., *H. oligactis* and *Tripalea clavaria*) (Excoffon et al., 2011; Ngo et al., 2021), or insects (e.g., Coleoptera, Diptera, Heteroptera, Hymenoptera, and Lepidoptera) (Honěk, 1993), to name a few. For example, in Odonates, large body size has positive effect on mating rate, fecundity, longevity, and survivorship; hence, there is a general fitness benefit to large size for this order of insects (Sokolovska et al., 2000). In *Drosophila pseudoobscura*, longevity and viability as well as fecundity are positively affected by large body size, whereas in grasshoppers too (e.g., *Romalea microptera*), large size has positive effect on fecundity (Akman & Whitman, 2008; Tantawy & Vetukhiv, 1960).

By contrast, larger body size might also demand more energy for maintenance simply because of the extra tissue that needs to be maintained (the “energy demand” hypothesis) and larger individuals might actually do worse in limited conditions (Reim et al., 2006). According to this hypothesis, smaller individuals might need less energy for self‐support and can thus reproduce sooner, causing advantage over bigger size (Blanckenhorn, 2000). For instance, in water striders, the food availability threshold over which small males are still able to copulate is more permissive than in larger males (Blanckenhorn, 2005).

Untangling which of these two alternative hypotheses (the “energy reserve” or “energy demand” hypothesis) contributes to the body size—fitness trait correlations are difficult in most occurrences (e.g., taxa and sex) because both of these traits might be affected by common causes. For example, food availability affects both growth and condition; hence, the positive relationship between size and fitness might in fact represent variation in resource availability (Gori et al., 2013; Reznick et al., 2000; van Noordwijk & de Jong, 1986; Yom‐Tov et al., 2006) and not direct consequences of large size or high growth per se. Moreover, in animals with larger size and longer life span, the study of body size effects in a laboratory is unfeasible. Furthermore, body size shows substantial pleiotropic effects on other traits. For instance, large size entails better fecundity, but may cause higher mortality later, because of the reproduction/survival trade‐off (Kirkwood & Rose, 1991; Sebestyén et al., 2020).

Here, we used the freshwater cnidarian *Hydra oligactis* (Pallas, 1766) as a model system to understand direct effects of body size on fitness traits. *Hydra* have remarkable regeneration abilities, which allow tissue excision and grafting between individuals, such that the body column of individual polyps can be experimentally increased or reduced. Previously, we have shown that these size changes are associated with altered sexual development time, fecundity, and postreproductive survival (Ngo et al., 2021). However, the reasons for these effects are still to be explained. To address this gap, we surgically manipulated *Hydra* body size, and the size‐manipulated individuals were subjected to different food availability environments during their sexual development. We hypothesized that the effect of body size should depend on the availability of food in a way that would reveal whether the “energy reserve” or the “energy demand” hypotheses are involved in explaining body size effects in *Hydra*. Specifically, if the “energy reserve” hypothesis is true, then larger animals will have better reproduction, such as earlier start and higher fecundity and/or postreproductive survival, because of the accumulated energy reserves, even in limited conditions. Conversely, if the “energy demand” hypothesis is correct, then large animals will do worse in reproduction and/or survival, because of the higher maintenance costs especially under limiting conditions.

## 2 MATERIALS AND METHODS

### 2.1 Study system

*Hydra oligactis*, commonly known as Brown Hydra, is a freshwater, gonochoristic invertebrate species, which is capable of asexual and sexual reproduction. It lives in highly seasonal habitats and reproduces by budding (asexually) in the major part of the year and starts to reproduce sexually at the onset of winter, when temperature is getting lower. After the cold stimulus, male polyps produce testes, while female polyps produce ovaries (about 2 weeks of difference between sexes) (Burnett & Reisa, 1973; Schuchert, 2010; Tökölyi et al., 2021; Quinn et al., 2012). Sexual reproduction results in the production of resting eggs that can survive harsh conditions, such as freezing. After sexual reproduction, a substantial proportion of polyps die in the next few months, when they go through a senescence‐like degradation (Tökölyi et al., 2017; Yoshida et al., 2006). This postreproductive senescence in *H. oligactis* is associated with reduction in the number of interstitial stem cells, a decreased ability to catch food, decline in tactile movements, decline in body size, and increase in mortality rate (Martínez & Bridge, 2012; Sebestyén et al., 2018; Tökölyi et al., 2017).

### 2.2 Hydra strains

All strains used for the experiment originate from one animal each from East Hungary. Strain X11/14 (female) and C2/7 (male) were established from two polyps collected from Tiszadorogma (47.6712°N, 020.8641°E) in September 2016, from a floodplain lake of Tisza river. Strain T3/1 (female) and T3/2 (male) originate from Tiszalúc (48.03420°N, 02.07894°N) an oxbow lake of Tisza river, in summer 2020. M26/9/10 (female) strain derives from river Hortobágy (47.57805°N, 021.14587°E), collected in summer 2020. Strain M83/4 (male) is from Gávavencsellő (48.17484°N, 021.61385°E); it was collected from an oxbow lake linked to Tisza river, in spring 2020. In all cases, *Hydra* polyps were collected from free‐floating and submerged macrophytes (e.g., *Ceratophyllum demersum, Ceratophyllum submersum, Myriophyllum spicatum, Stratiotes aloides, and Nuphar lutea*), and all of the collected polyps were multiplied to strains by asexual budding and were maintained asexually under standard conditions in the laboratory since their collection (standard feeding, temperature, and light–dark cycle, see below).

### 2.3 Experimental maintenance

Animals were kept individually in six‐well cell culture plates with 5‐ml standard *Hydra* medium per well (*Hydra* medium composition: 1 mM Tris, 1 mM NaCl, 1 mM CaCl_2_, 0.1 mM KCl, 0.1 mM MgSO_4_; pH: 7.6 (Tökölyi et al., 2017)). They were fed individually with 20 μl fresh *Artemia nauplii* suspension using an automatic pipette (FinnPipette). During feeding, the number of gonads (testes and eggs) and the number of detached buds were recorded, and about 2 h after feeding, the animals were moved to clean *Hydra* medium. For the experiment, 3‐week‐old animals cultured on 18°C degrees with 12/12 h' light/dark cycle (simulating summer conditions) were randomly selected and paired with similar individuals from the three female and three male strains before the experimental treatments.

### 2.4 Size manipulation treatment

For the size manipulation treatment, tissue grafting was used to produce individuals with enlarged, control, and reduced body size with randomly picked individuals from the six strains. Two individuals were selected from the same strain and paired randomly, then a ring shaped tissue was cut from both *Hydra* polyps' body column. For the “enlarged‐reduced” pair, we cut rings differing in size and then switched these rings between the animals (the enlarged animal received a big ring in return for the small one that was cut out; the reduced animal received a small ring in return for the big one that was cut out). As a result, we obtained animals with enlarged and reduced body size. For the “control–control” pair, rings approximately identical in size were switched between the paired polyps. The head, the ring, and the foot region were put together in the correct order and strung up on a glass microcapillary needle until the pieces stuck together (which took about 1–2 h). Then, we removed the polyps from the needle and left them to heal until the next day (see the procedure in: Ngo et al., 2021).

### 2.5 Food availability manipulation

The day after size manipulation, experimental animals were moved to a cooled incubator (Pol‐Eko ST2) set to 8°C degrees with 8/16 h' dark/light cycle (simulating winter conditions) to induce sexual reproduction by cold stimulus (first day of experiment). To examine the effect of food availability on the different body sizes, three feeding groups were made with the size‐manipulated animals. Food manipulation was performed immediately after cooling, when animals started gonadogenesis. The first group was starved for 2 weeks (low food, 0×). The second group received food two times per week for 2 weeks (normal or medium food, 2×), and the third group received food four times per week (high food, 4×) for 2 weeks. All experimental animals were fed individually (hence, no food competition was involved) with 20 μl fresh *Artemia nauplii* suspension, as detailed in the section *Experimental maintenance*. After the 2‐week food treatment, all groups were fed two times per week until the end of the experiment, which was 22 weeks after cooling.

**FIGURE 1:**
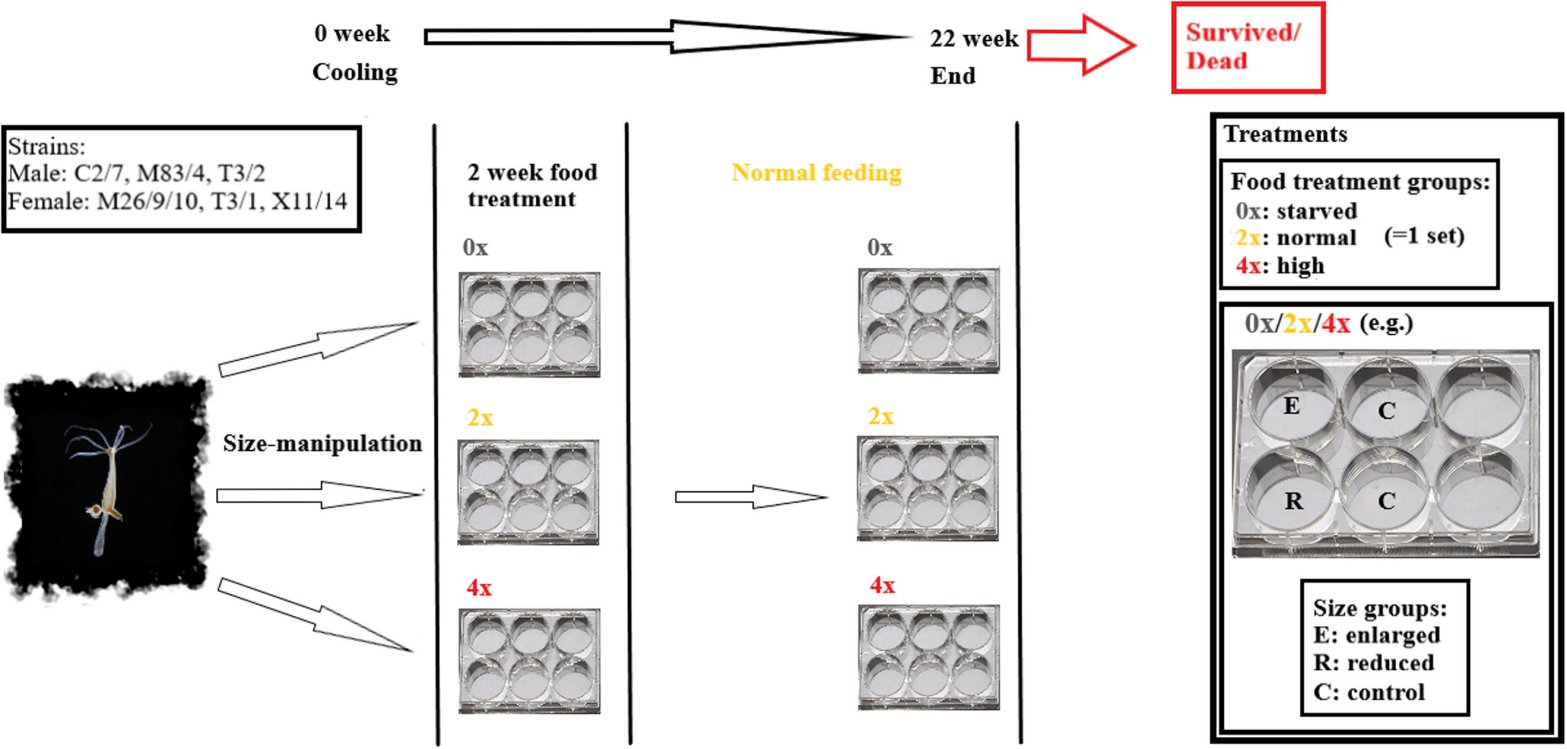
From the six strains (C2/7, M83/4, T3/2, M26/9/10, T3/1, and X11/14), individuals were randomly selected/paired and after the size manipulation treatment (E‐enlarged, R‐reduced, C‐control), polyps were assigned to one of the three food treatment groups (0×‐starved, 2×‐normal, 4×‐high). In the first 2 weeks, polyps were fed according to their feeding group, after 2 weeks of food treatment, all animals were fed normally (2×) until the end of the study. Finally, the animals' final status was defined as survived or dead.

Throughout the study, animals were maintained in sets, which contained one plate/feeding group (0×, 2× or 4×) with four size‐manipulated animals (one “enlarged‐reduced” pair and a “control–control” pair” or two “enlarged‐reduced” pairs; Figure 1). The individuals' location in the plate was randomly chosen, and the three feeding treatment plates' sequence in the stock was randomized too. We aimed to have 16 animals in each strain and group (e.g., strain: C2/7/food treatment: starved/size manipulation: reduced).

For the experiment, 864 individuals (12 sets/strain = 144 polyps/strain) were used in total, and 820 remained after failed (e.g., the body parts did not attach after size manipulation), sex‐changed, and hermaphrodite animals were taken out of the analysis. The exact number of polyps in each strain and experimental groups are shown in Table 1.

**TABLE 1:** Exact sample size in each strain (divided into male (C2/7, M83/, T3/2) and female M26/9/10, T3/1, X11/14 strains) and experimental group (divided into food treatment and size manipulation).

Male strains									
Strain	C2/7 *N* = 142			M83/4 *N* = 131			T3/2 *N* = 140		
Food treatment	0× *N* = 48	2× *N* = 48	4× *N* = 46	0× *N* = 43	2× *N* = 16	4× *N* = 42	0× *N* = 48	2× *N* = 46	4× *N* = 46
Size manipulation	E = 16	E = 16	E = 14	E = 15	E = 15	E = 14	E = 16	E = 15	E = 14
	R = 16	R = 16	R = 16	R = 13	R = 16	R = 14	R = 16	R = 15	R = 16
	C = 16	C = 16	C = 16	C = 15	C = 15	C = 14	C = 16	C = 16	C = 16

Female strains									
Strain	M26/9/10 *N* = 139			T3/1 *N* = 134			X11/14 *N* = 134		
Food treatment	0× *N* = 47	2× *N* = 46	4× *N* = 46	0× *N* = 45	2× *N* = 47	4× *N* = 42	0× *N* = 47	2× *N* = 43	4× *N* = 44
Size manipulation	E = 16	E = 15	E = 15	E = 15	E = 16	E = 14	E = 15	E = 16	E = 15
	R = 15	R = 15	R = 15	R = 16	R = 15	R = 14	R = 16	R = 13	R = 14
	C = 16	C = 16	C = 16	C = 14	C = 16	C = 14	C = 16	C = 14	C = 15

*Note*: In the food treatment “0×” is starved, “2×” is normally and “4×” is a highly fed group, next to it, the number of individuals in each group. In the size manipulation experimental group, “E” is enlarged, “R” is reduced and “C” is control animals' number.

### 2.6 Data recording

Timing of sexual reproduction (first mature egg on females and first mature testis on males), and the number of gonads (total number of eggs in females and maximum number of testes in males) were recorded four times per week under a binocular stereo microscope (Euromex StereoBlue). Sexual development time is defined as the number of days elapsed after lowering temperature until gonads were clearly visible on polyps for the first time. In order to record the number of eggs on female individuals, we counted the eggs on them, as well as the detached eggs in the well. After gametogenesis, the number of detached eggs was summed up (unfertilized eggs detach from the polyp after some time), to obtain total egg production. To record the number of testes on a male individual, we laid it on its side and counted the clearly visible testes. In males, the maximum number of testes was used as a proxy for fertility, because the number of testes are changing from the first day of gametogenesis to the last day (there is an increase in the early stages until they reach a maximum, thereafter, when the individual start to go through senescence, there is a decrease in the number of gonads). Testes number was used as a proxy for fertility in males, because sperm counting is difficult. We expect higher sperm number, consequently higher fertility from a polyp with more testes.

**FIGURE 2:**
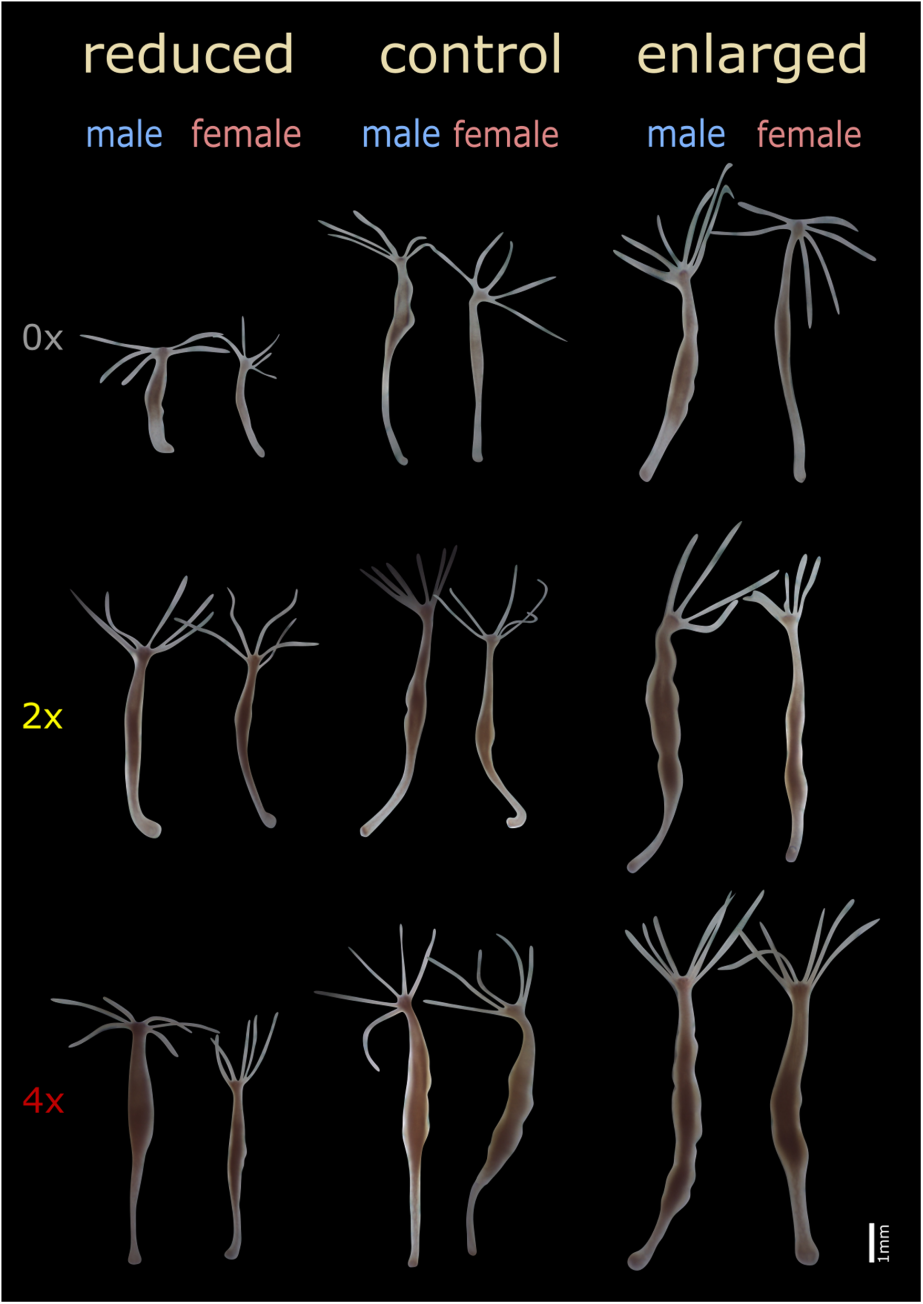
Male and female hydra polyps after 2 weeks of food treatment in the reduced, control and enlarged size‐manipulated groups. First row is the starved (0×), second is the group which was fed two times per week (2×), and the last is the highly fed (4×) group. In the three columns (reduced, control, and enlarged), the first animal depicted is always male and the second one is always female.

Photographs of the animals were taken after size manipulation, but before cooling to quantify size change as a result of size manipulation and after the 2 weeks of food treatment to see its effect (Figure 2). We used a CMEX microscope camera to photograph animals under a stereo microscope. Photographs of the individuals were taken on 1 mm grid paper, and the program ImageJ (Schneider et al., 2012) was used for measuring the area of the body column from which body size was calculated (expressed as the area of the polyps in pixels, divided by the square length of standard millimeter in pixels).

Finally, the animals' final status was recorded on the last day of experiment (22 weeks after cooling). The individual was scored as regenerated/survived if the polyp looked healthy (intact tentacles/eating, tactile movements, and orange color), and/or it made a new bud after sexual reproduction and if it did not reproduce sexually (asexual), but stayed alive until the last day. The animals were scored as dead if they disintegrated or consisted only of necrotic tissue on the last day of experiment.

### 2.7 Statistical analysis

To test the interactive effects of body size and food availability on life history traits, we used Generalized Linear Mixed Models (GLMMs), as implemented in the glmmTMB v1.1.4 package in R v4.2.1 (Brooks et al., 2017; R Core Team, 2022). As dependent variables, we used sexual development time (after experimentally lowering the temperature), fecundity (total no. detached eggs in females, and maximum no. testes for males), and survival. Models were fitted separately for female and male strains with strain ID as a random effect. Size manipulation pair ID was included as an additional random effect to take into account the fact that individuals exchanging body parts might be more similar to each other than expected by chance.

We considered GLMMs with Gaussian, Poisson, or Negative Binomial distribution (with quadratic or linear parametrizations; Brooks et al., 2017) to analyze sexual development time and fecundity. The type of distribution was selected based on model comparisons with Akaike's information criteria corrected for small sample size (AICc). Based on this, sexual development time and the number of testes produced in males were analyzed with Gaussian GLMMs, while a Negative Binomial model with quadratic parametrization ranked best to analyze the number of eggs produced by females. Where needed, we explicitly modeled heterogeneity of variance using the *dispformula* argument in glmmTMB. Finally, survival was analyzed with GLMMs with binomial distribution. Model diagnostics were performed with the DHARMa package v0.4.5 (Hartig, 2022), checking for approximate normality of residuals, homogeneity of variance, and the presence of outliers. For each model, we fitted a full model with size manipulation group, food treatment group, and their interaction as fixed effects (in addition to strain and size manipulation pair ID as random effects). From these full models, we performed stepwise model simplification followed by Likelihood Ratio Tests (LRTs) to remove nonsignificant predictors. Graphs were produced with the *ggplot2* package v3.3.6 (Wickham, 2016).

## 3 RESULTS

### 3.1 Size manipulation

**FIGURE A1:**
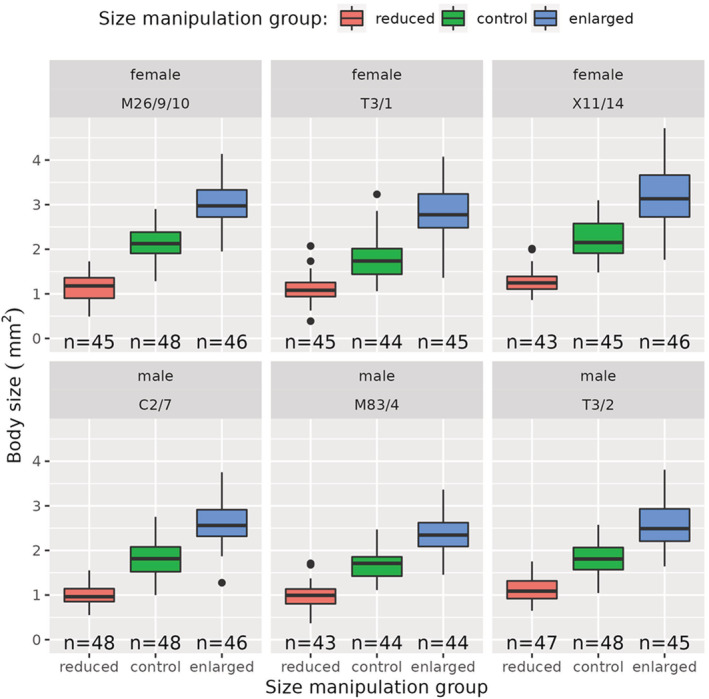
Body sizes after size manipulation in the three female (M26/9/10, T3/1, X11/14) and three male (C2/7, M83/4, T3/2) strains. Reduced (red box), control (green box) and enlarged (blue box) polyps' sizes on the *y*‐axis in mm^2^.

Size manipulation successfully increased or decreased the size of experimental polyps in enlarged and reduced animals relative to controls (Gaussian GLMM, *χ* ^2^ = 949.790, *p* < .001). Reduced animals were approximately 50% smaller than controls, while enlarged animals were about 50% larger (Figure A1).

### 3.2 Sexual development time

**FIGURE 3:**
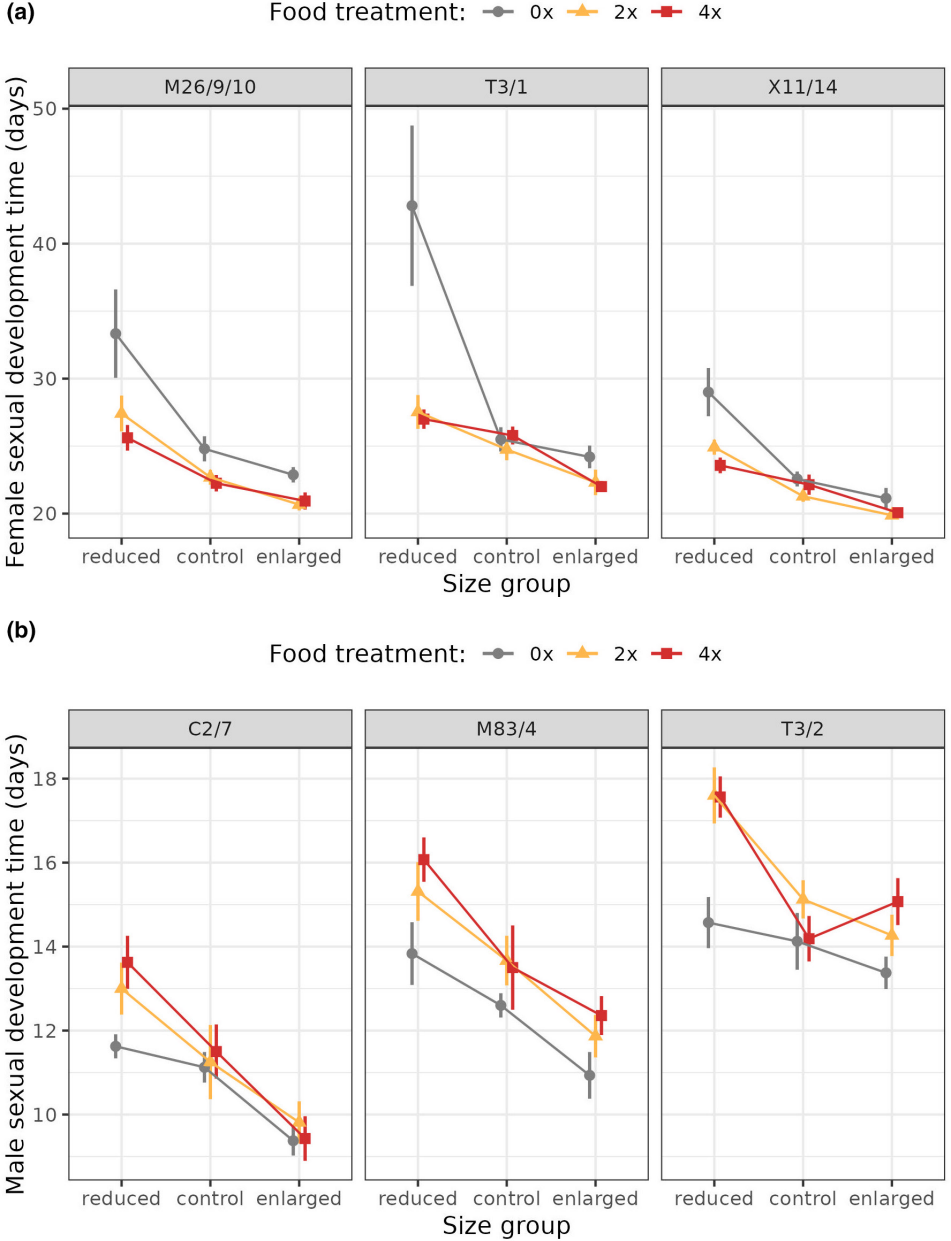
Time required to develop the first gonads after lowering the temperature in three female strains (M26/9/10, T3/1, X11/14; a) and three male strains (C2/7, M83/4, T3/2; b) of *H. oligactis* exposed to body size manipulation and kept under three different feeding regimes (0×, 2×, 4×; see Figure 1 for experimental design). Female hydra polyps with experimentally reduced body size have a longer sexual development time than individuals with an enlarged or control size. The effect of body size depends dramatically on resource availability during gonad development, such that starved small polyps produce eggs latest. Male hydra polyps with experimentally reduced body size have a longer sexual development time than individuals with an enlarged or control size. However, the effect of body size depends on resource availability during gonad development, such that the effect of size manipulation is higher in individuals that received more food.

In female strains, the time required to produce the first gonads decreased with increasing body size and with the amount of food received (Figure 3a). However, the effect of body size depended dramatically on resource availability during gonad development, such that enlarged and 4× fed individuals started sexual reproduction the earliest, while reduced polyps delayed their reproduction substantially more, especially if they also received less food (significant interaction between body size and food availability on sexual development time; Gaussian GLMM, *χ* ^2^ = 21.154, *p* < .001; Figure 3a). In all three strains, the first eggs were produced on average 20–28 days after lowering the temperature, but reduced and starved polyps needed 29–43 days on average to produce the first eggs, with strain T3/1 delaying reproduction most (Figure 3a).

In male animals, enlarged and starved individuals started sexual reproduction earliest, but the effect of size manipulation was higher in individuals that received more food (significant interaction between body size and food availability on sexual development time; Gaussian GLMM, *χ* ^2^ = 13.051, *p* = .011; Figure 3b). The three strains differed in their sexual development time: polyps in strain C2/7 started sexual reproduction earliest (about 10–14 days after the cold stimulus), while those in strain T3/2 developed gonads the latest (about 13–18 days after the cold stimulus), with strain M83/4 being intermediate between the two (Figure 3b). However, despite the differences in timing, reduced animals advanced their reproduction more when they were also starved in all three strains (Figure 3b).

### 3.3 Fecundity

**FIGURE 4:**
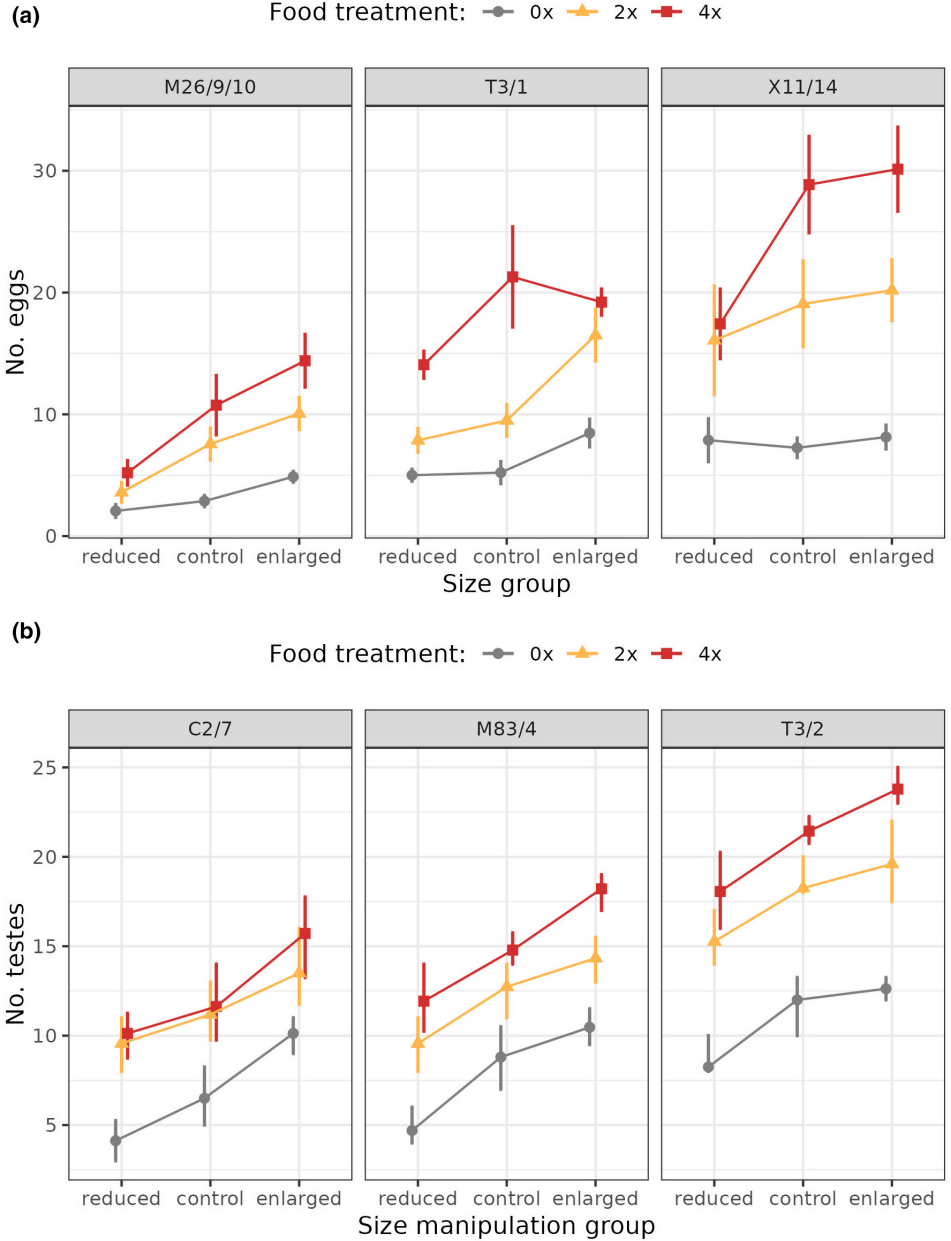
Number of eggs (a) and number of testes (b) produced by hydra polyps exposed to body size manipulation and kept under three different feeding regimes (0×, 2×, 4×; see Figure 1 for experimental design). Female hydra polyps with enlarged body size and receiving more food produce a higher number of eggs, but there is no interaction between food and size treatments. Likewise, male hydra polyps with enlarged body size and those receiving more food produce a higher number of testes, but there is no interaction between food and size treatments.

In females, enlarged and fed individuals produced the most gonads. There was no significant interaction between size and food treatments (Negative Binomial GLMM, *χ* ^2^ = 4.771, *p* = .312). However, both the size and the food treatments significantly affected the number of eggs produced (size treatment *χ* ^2^ = 57.855, *p* < .001; food treatment *χ* ^2^ = 110.260, *p* < .001; Figure 4a). The three strains differed in overall egg production (about 2–15 eggs produced/polyp in strain M26/9/10; 5–20 in T3/1 and 6–32 in X11/14), but the response to treatments was similar across strains.

In males, enlarged and fed individuals produced the most gonads, but the interaction between size and food treatments was not significant just like in the female strains (Gaussian GLMM, *χ* ^2^ = 8.334, *p* = .080). However, separately, both the size and the food treatments significantly affected the number of testes produced (size treatment *χ* ^2^ = 242.310, *p* < .001; food treatment *χ* ^2^ = 243.370, *p* < .001; Figure 4b). The three strains differed in overall testes production (on average, 4–16 testes/polyp in C2/7, 5–18 in M83/4 and 2–26 in T3/2, depending on treatment), but all strains responded similarly to treatments.

### 3.4 Survival

**FIGURE 5:**
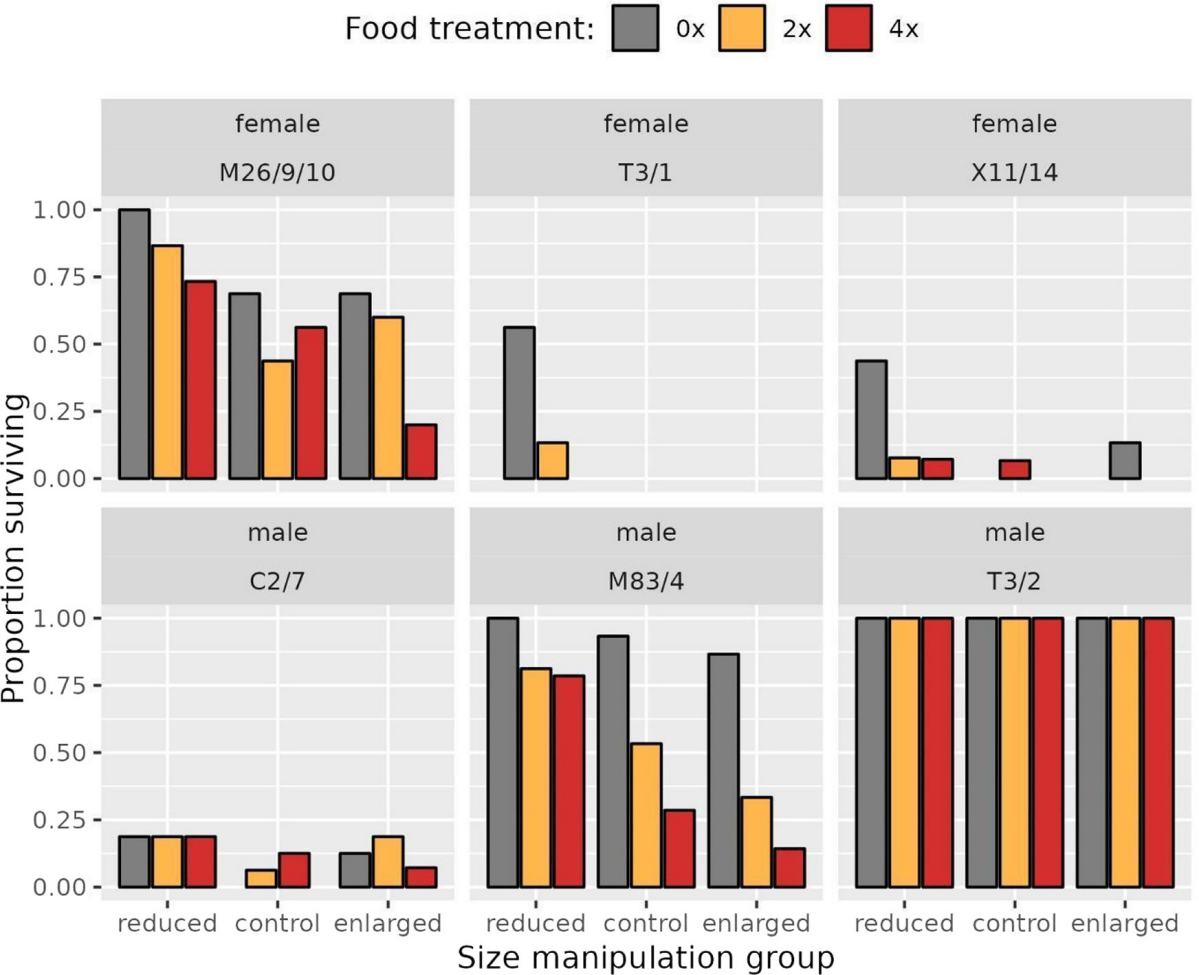
Both size manipulation and resource availability had a significant effect on survival, such as the reduced and starved animals' survival rate was the highest, but there was no interaction between the two treatments in affecting survival rate. There are exceptions in male strains C2/7, T3/2 and female strain X11/14, where the highest survival rate is not in the starved and reduced groups. Male polyps (C2/7, M83/4, T3/2) had higher survival rate, than the females (M26/9/10, T3/1, X11/14).

There was no interaction between the two treatments in affecting survival rate (Binomial GLMM, *χ* ^2^ = 7.532, *p* = .110), but both size manipulation and resource availability had a significant effect on survival, such that survival rate was highest in reduced and starved animals (Binomial GLMMs, size manipulation: *χ* ^2^ = 64.364, *p* < .001; food treatment, *χ* ^2^ = 44.219, *p* < .001; Figure 5). Total survival rate of the six strains was highly variable (female strains: M26/9/10 = 64%; T3/1 = 8.2%; X11/14 = 8.9%; male strains: C2/7 = 12.6%; M83/4 = 63.3%; T3/2 = 100%). All of the polyps survived in strain T3/2 regardless of their size and food treatment. By contrast, in the female strain T3/1, which had the highest postreproduction mortality rate, just 11 polyps survived out of 134 (8.2% survival rate), and all of them were reduced animals.

## 4 DISCUSSION

Body size should have complex effects on the energetic balance of an organism, with large size either acting as a reserve or as an extra cost to be expended (Blanckenhorn, 2000; Blanckenhorn et al., 1995). Here, we investigated this energy balance mechanism by applying experimental body size manipulation in freshwater *Hydra* to obtain artificially created size variation, allowing us to investigate the direct effects of body size, independent of the past environmental conditions and life history experienced by these animals. We combined size treatment with three distinct resource levels during the sexual development period. Based on both of our hypotheses, we expected interactions between body size and food availability in their effect on reproduction and/or survival. Specifically, we expected that large animals will be less affected by reduced resources if the “energy reserve” hypothesis is true, because larger body size could entail more energy reserves that can be allocated to different life history components even under limiting conditions (Lindstedt & Boyce, 1985; Roff, 1993; Stearns, 1992). By contrast, we expected large animals to be more affected by variation in resource availability if the “energy demand” hypothesis is true, because large size would demand more energy for maintenance, and this extra energy demand could be more easily satisfied under high but not low resource conditions (Blanckenhorn, 2000, 2005; Reim et al., 2006). Although we treated the two hypotheses as separate mechanisms, it has to be emphasized that energetic costs and benefits of large size might be just two facets of the same mechanism (i.e., energy balance) to which resource availability also integrates, albeit providing shorter term contributions. Hence, a clear separation of size‐ and resource availability effects might not be entirely possible.

With this limitation in mind, we found that sexual development time depended on food availability more strongly in small than in large individuals, consistent with an “energy reserve” effect of body size. On the contrary, we did not find any food‐dependent effects of body size on fecundity or survival. We discuss these findings in turn below.

### 4.1 Sexual development time

Larger females produced gonads earlier, consistent with our previous observations (Ngo et al., 2021). The accelerating effect of body size dramatically depended on food treatment, as enlarged, highly fed individuals produced eggs earliest and diminished, starved ones latest, requiring about one more week to produce their first eggs.

Larger (enlarged and control) males also started to reproduce earlier, with the size manipulation effect amplified at high food availability. However, in contrast to females, starved male polyps started reproduction earlier than well‐fed ones, regardless of their body size, this effect was most pronounced on low food (i.e., starved, reduced polyps started reproduction first).

These results support the “energy reserve” effect of large size, since sexual development time was affected by both body size and resource availability, and the effect of resource availability was lowest in enlarged individuals. Surprisingly, however, the effect of food availability differed in the two sexes: lack of food delayed reproduction in females, but accelerated reproduction in males. Several explanations could lie behind these sex differences. Most importantly, sexual reproduction might be more expensive for females, since eggs are thought to be more costly to produce than sperm (Hayward & Gillooly, 2011). As a result, females should be impacted most in the reduced‐starved group because they lack both internal (body size) and external (food) reserves. However, while this hypothesis can explain the stronger effect observed in females, it cannot account for small males responding the opposite way to resource shortage. A potential explanation could be that, besides their lower energy need for gamete production (Hayward & Gillooly, 2011), males may interpret low resource availability as an additional cue for starting sexual reproduction (Burnett & Reisa, 1973; Tökölyi et al., 2021). Hence, the stronger the intensity of cues signaling the approach of winter (low food and reduced temperature) may cause earlier start of reproduction regardless of their size. Thus, males might be differently affected by the interaction between body size and resource availability, because they must not fall behind in sperm production to other competing males, since *Hydra* eggs are not fertilizable for a long time (Kaliszewicz & Lipińska, 2012; Littlefield et al., 1991; Tökölyi et al., 2021). Taken together, large body size may well physiologically constitute a net long‐term energy reserve for both male and female *Hydra* to be invested in reproduction, thus speeding up their gonad development. By contrast, short‐term food (i.e., energy) supply further accelerates reproduction (i.e., has the same effect as long‐term energy reserves) in females but has the opposite effect in males, likely because males invest not in more sperm (testes) reproduction but into other, more long‐term life history traits such as, for example, growth or longevity (because they already have enough cheap sperm) (Blanckenhorn, 2005; Reim et al., 2006).

### 4.2 Fecundity

The number of gonads increased additively with body size (reflecting the larger energy reserve of enlarged animals) and with food (reflecting the larger energy influx), in both sexes. This pattern can be observed in most insects, birds, mammals, or reptiles too (Allainé et al., 1987; Ford & Seigel, 1989; Honěk, 1993). We had expected interactive effects between body size and resource availability on the number of gonads, such that larger individuals would show a smaller fecundity difference on low vs. high food if the “energy reserve” hypothesis is true and a larger fecundity difference according to the “energy demand” hypothesis. However, we did not find such an interaction.

One potential explanation behind this lacking interaction could be the altered reproductive timing of individuals with reduced body size. Reduced and starved females produced their first egg about 1 week later than all other experimental groups. This apparently allowed them to accumulate additional reserves from their food to increase their fecundity, effectively canceling any interaction effect. A longer starvation period (or reduced feeding after the treatments) might have elicited a stronger effect, although it is possible that the reduced and starved polyps would have delayed their reproduction even further. Thus, regardless of whether energy reserves were limited via body size (long‐term) or directly via food supply (short‐term), a presumed fecundity target was adjusted by delaying reproduction rather than by producing fewer eggs earlier.

Adjustment in reproductive timing, however, cannot fully explain a lack of interaction between food and size treatments on fertility in males. Reduced and starved males advanced their reproduction, yet their fertility was not more negatively affected. Two factors might explain this surprising finding. First, the reproductive advancement in the smaller, reduced males was merely 3.5 days at 4× feeding, while it was 2 days at 0× feeding conditions. This small difference might not be sufficient to generate an observable effect on fertility. Second, our measurement of male fertility might not be precise enough to detect such small effects, since we counted gonads visible to the naked eye, whereas these gonads might differ in size and/or sperm production. Future studies will require assessing male fertility more precisely, for example, via reproductive cell counts or reproductive gene expression assays.

### 4.3 Survival

Consistent with a previous study in *Hydra* (Ngo et al., 2021), reduced polyps showed enhanced survival. The present study tested a larger number of *Hydra* strains so we could generalize the body size–longevity relationship in *Hydra*. Similar negative relationship between body size and longevity is seen across breeds of a number of domesticated animals (e.g., dogs and horses; Austad, 2010; Bartke, 2017; Kraus et al., 2013; Rollo, 2002). We hypothesize that the higher survival of small polyps is due to their lower investment in reproduction, so more resources could be shunted into survival (Kirkwood & Rose, 1991; Sebestyén et al., 2020). However, we again did not observe an interaction between body size and food availability on survival, which could be explained as the lacking of interaction in the fecundity analysis. Moreover, there was a big difference between the strains in the number of survived animals, some of them showing 100% survival, and others almost complete mortality. These large deviations certainly reduced the power of our analysis by obscuring any treatments effects. These different survival rates of the strains are currently hard to predict. Genetic differences among our strains are likely to be low (Miklós et al., 2021), and the laboratory conditions under which they were maintained were identical. However, they might differ in the composition of their associated microbiota (something that we currently do not know), and microbiota composition has strong effects on *Hydra* physiology (reviewed in: Taubenheim et al., 2022). This is all the more likely because we here detected a substantial drop in the survival rate of strain C2/7 relative to our previous study (Ngo et al., 2021). Since nothing else changed in our experimental conditions, we suspect that these animals might have experienced a change in the composition of host‐associated microbes, although we cannot currently verify this assumption. Future studies should take into account the presence of specific host‐associated microbes as well.

We also found that starved individuals had a higher survival rate than highly fed ones. This pattern is very similar to the dietary restriction effects observed in a number of different animal species (Magwere et al., 2004; Moatt et al., 2016), and it could be also explained by an altered reproduction/survival trade‐off in both sexes (Adler et al., 2016; Kirkwood & Rose, 1991; Sebestyén et al., 2020). Interestingly, we did not find such a dietary restriction effect in a previous study performed on the same *Hydra* species (Tökölyi et al., 2017). However, there are several differences among this and our previous study. First, in the current experiment, we applied a 2‐week‐long intense starvation, whereas animals in our previous study were exposed to a continuous low food supply. Second, and perhaps more importantly, the strains in the previous study were different and they had a very high overall survival rate, similar to strain T3/2 presented here. This high survival rate might have precluded detecting any increase in survival in response to food reduction. We therefore conclude that there appears to be a dietary restriction effect on survival in *Hydra*, with strains with a high overall survival rate merely less affected.

To conclude, we found support for the hypothesis that body size acts as an energetic buffer in *Hydra*, enabling large individuals to achieve sexual development at the same speed largely independent of the current environmental conditions. Small polyps with long‐term energy reserves under a presumed threshold, by contrast, plastically adjusted their reproductive timing to food shortage. Likely as a consequence, any effects of body size on fecundity and survival did not depend on food availability. We thus did not find strong support for an energetic cost of large body size, since animals with an enlarged body size did not experience a stronger reduction in fitness traits under resource shortage than small individuals. This lacking “energy demand” effect might be specific to ectothermic animals that require much less energy for tissue maintenance than endothermic species (Gillooly et al., 2001). In general, it is difficult to experimentally calibrate short‐term (i.e., food supply) and long‐term (i.e., body size) energy reserves supply around the putative individual physiological energetic thresholds mediating their reproductive life history responses (Blanckenhorn et al., 1995). Experimental size manipulation might address that question in the future by exposing size‐manipulated *Hydra* to increased tissue maintenance costs (e.g., by experimentally increasing temperature).

## AUTHOR CONTRIBUTIONS

**Réka Gergely:** Conceptualization (supporting); data curation (lead); funding acquisition (equal); investigation (lead); methodology (equal); project administration (lead); visualization (equal); writing – original draft (equal); writing – review and editing (equal). **Jácint Tökölyi:** Conceptualization (lead); formal analysis (lead); funding acquisition (equal); methodology (equal); project administration (supporting); visualization (equal); writing – original draft (equal); writing – review and editing (equal).

## CONFLICT OF INTEREST

All authors declare that there is no conflict of interest.

## Data Availability

Data: Gergely, Reka; Tokolyi, Jacint (2022): data_resource_availability_modulates_the_effect_of_body_size_on_reproductive_development.xlsx. figshare. Dataset. https://doi.org/10.6084/m9.figshare.21428130.v1. Code: https://github.com/jtokolyi/Hydra‐SizeFoodInteraction.
